# Role of right dorsolateral prefrontal cortex–left primary motor cortex interaction in motor inhibition in Parkinson’s disease

**DOI:** 10.3389/fnagi.2025.1524755

**Published:** 2025-03-05

**Authors:** Zhen Wang, Jianing Wei, Yuyu Song, Yuting Li, Yin Wu, Robert Chen, Zhen Wang, Jian Zhang, Xiaoyin Tan, Ke Liu

**Affiliations:** ^1^School of Sport and Health Science, Xi'an Physical Education University, Xi'an, China; ^2^School of Psychology, Shanghai University of Sport, Shanghai, China; ^3^Henan University of Science and Technology Sports Institute, Luoyang, China; ^4^Department of Chinese Medicine Nursing, School of Nursing, Anhui University of Chinese Medicine, Hefei, China; ^5^School of Economics and Management, Shanghai University of Sport, Shanghai, China; ^6^Krembil Research Institute, University Health Network, Toronto, ON, Canada; ^7^Division of Neurology, Department of Medicine, University of Toronto, Toronto, ON, Canada; ^8^School of Martial Arts, Shanghai University of Sport, Shanghai, China; ^9^Faculty of Health Sciences and Sports, Macao Polytechnic University, Macau, Macao SAR, China; ^10^Shanghai Punan Hospital of Pudong New District, Shanghai, China

**Keywords:** Parkinson’s disease, motor inhibition, interhemispheric interaction, dorsolateral prefrontal cortex, pre-supplementary motor area

## Abstract

**Background:**

Impaired motor inhibition in Parkinson’s disease (PD) is associated with functional alterations in the frontal-basal ganglia (BG) neural circuits. The right dorsolateral prefrontal cortex (DLPFC), pre-supplementary motor area (pre-SMA), and primary motor cortex (M1) play key roles in regulating this inhibition. However, the changes in interhemispheric interactions during motor inhibition in PD have not been clearly defined.

**Methods:**

We used dual-site paired-pulse transcranial magnetic stimulation (ppTMS) to examine the interactions between the right DLPFC and pre-SMA and the left M1 in 30 patients with early-stage PD and 30 age-matched healthy controls (HC) during both resting and active conditions, specifically while performing a stop-signal task (SST).

**Results:**

Stop-signal reaction times (SSRT) were significantly longer in PD patients compared to HC. The right DLPFC–left M1 interaction, at both short- and long-latency intervals, showed enhanced inhibition in PD following the stop-signal. In PD patients, SSRT was correlated with the inhibition of the right DLPFC–left M1 interaction, with stronger inhibition associated with shorter SSRT.

**Conclusion:**

The deficit in reactive inhibition observed in PD is linked to an abnormal modulation of the right DLPFC–left M1 interaction during the stopping process.

## 1 Introduction

Motor inhibition involves the cancellation of dominant or inappropriate responses and requires coordination within the sensorimotor system. It can be categorized into reactive inhibition and proactive inhibition. Reactive inhibition refers to the ability to immediately halt an ongoing action upon receiving a stop signal, whereas proactive inhibition involves adjusting motor strategies in anticipation of future actions based on the situation (Aron, 2011). In Parkinson’s disease (PD), inhibition deficits become more pronounced as the disease progresses (Gauggel et al., 2004; Obeso et al., 2011; Mirabella et al., 2012), affecting an individual’s ability to pursue future-oriented goals (Di Caprio et al., 2020). However, the underlying neural mechanisms contributing to motor inhibition deficits in PD are not yet fully understood.

Changes in the frontal-basal ganglia network was associated with changes in motor inhibition (Coxon et al., 2012). The primary motor cortex (M1), as the main output structure of this circuit, is crucial in “braking” movement output during motor inhibition (Berardelli et al., 2008; Wu et al., 2011a). The frontal cortex, particularly the pre-supplementary motor area (preSMA) and the dorsolateral prefrontal cortex (DLPFC), play an important role in motor inhibition (Trujillo et al., 2019). When halting an already - initiated action, the pre-SMA and right inferior frontal gyrus send a stop signal through the BG network to intercept prepotent response, inhibiting BG output and resulting a global inhibitory effect on M1 (Aron et al., 2007; Badry et al., 2009). However, patients with PD commonly exhibit dysfunction and reduced activity in the supplementary motor complex (SMC) (Rahimpour et al., 2022), especially in pre-SMA (Herz et al., 2014). As a functional region essential for inhibiting competitive motor processes, damage to the pre-SMA can lead to impaired motor inhibition when there is competition between movements (Nachev et al., 2007). Effective connectivity analysis indicates that prefrontal regions, including the DLPFC, modulate task-specific activity by targeting the motor cortex, ultimately leading to motor cessation (Apšvalka et al., 2022). The right DLPFC is significantly related to inhibition control, and its activity changes can be used as an indicator of an individual’s motor inhibition ability (Hung et al., 2018). In the ON-condition, a higher regional blood flow in the right DLPFC, pre-SMA and M1 is predictive of better inhibitory control performance, especially in improving the reaction time of PD patients (Obeso et al., 2013; Criaud et al., 2016; Trujillo et al., 2019). However, whether behavioral performance during PD motor inhibition is regulated by DLPFC and pre-SMA interhemispheric interactions with M1 remains unclear.

Dual-site paired-pulse transcranial magnetic stimulation (ppTMS) is a technique that allows for the measurement of causal functional interactions between brain regions at a millisecond scale, under different movement states (Mars et al., 2009; Kroeger et al., 2010). Previous research has demonstrated that the interaction between motor-related cortical areas and contralateral M1 varies at different interstimulus intervals (ISI), which can be categorized as short-latency (6–10 ms) or long-latency (40–50 ms). This variation in cortical interaction reflects distinct cortico-cortical physiological pathways that regulate interhemispheric activity (Ni et al., 2009). In our study, we applied this method to examine cortico-cortical interactions from the right DLPFC and pre-SMA to the left M1 during motor inhibition, aiming to identify dynamic time windows that could indicate motor inhibition defects in PD.

Motor inhibition is commonly assessed using the stop-signal task (SST) (Logan et al., 1984). Building on our previous research, we selected the maybe stop task (MST) and never stop task (NST) to evaluate reactive and proactive inhibition performance (Wang et al., 2022b). In the NST, a go signal is never followed by a stop signal. In the MST, a stop signal may follow a go signal to cancel the individual’s response. The stop-signal reaction time (SSRT) refers to the response time to the stop signal in the MST, while the response delay effect (RDE) represents the difference in response times to the go signal between the NST and MST tasks. The SSRT is used to measure reactive inhibition, and the RDE is used to measure proactive inhibition (Pan et al., 2018).

In this study, we combined ppTMS with SST to observe changes in interhemispheric interactions at various time points following stimulus onset in patients with PD and healthy controls (HC). We hypothesized that interhemispheric interactions would be reduced, contributing to motor inhibition deficits in patients with PD.

## 2 Materials and methods

### 2.1 Participants

We studied 30 patients with idiopathic PD and 30 HC, matched for age, sex, and educational level. All participants were right-handed, as confirmed using the Edinburgh Handedness Inventory (Oldfield, 1971), and had normal cognition (Montreal Cognitive Assessment, MoCA scores ≥26) (Nasreddine et al., 2005). PD diagnosis was based on the Movement Disorder Society (MDS) clinical diagnostic criteria (2015), and patients were recruited from the neurology clinic of Punan Hospital, Pudong New Area, Shanghai, China. Patients underwent the MDS-Unified Parkinson’s Disease Rating Scale, Part III (MDS–UPDRS–III) assessment at the start of the study. All participants were physically independent (Hoehn & Yahr stages I–II) and had no other neurological disorders or impulse control disorders. Patients maintained their regular medication regimens throughout the study, and all tests were conducted during the medication-ON period. Written informed consent was obtained from all participants after the study details were explained. The study protocol was approved by the Shanghai University of Sport Ethics Committee (102772020RT107) and registered with the Chinese Clinical Trial Registry (ChiCTR2000038517).

### 2.2 TMS protocol

Dual-site ppTMS pulse was used to investigate interactions between the right DLPFC and pre-SMA to the left M1 during movement processing. Two 50 mm figure-of-eight coils (Alpha Branding Iron, Magstim) were connected to two Magstim 200 stimulators (Whitland, Dyfed, UK). The left and right M1 were defined as the locations where TMS induced motor evoked potentials (MEPs) with the highest peak-to-peak amplitude in the contralateral first dorsal interosseous (FDI) muscle at a given suprathreshold stimulator intensity. Resting motor threshold (RMT) was defined as the lowest TMS intensity required to generate MEPs greater than 50 μV in at least 5 of 10 trials, with the target muscle completely relaxed. The test stimulus (TS) coil was applied over the left M1 with the handle pointing backward at a 30–45° from the mid-sagittal line to produce a posterior–anterior directed current. The conditioning stimulus (CS) coil was placed over the right DLPFC or pre-SMA.

### 2.3 Electromyographic recordings

Surface electromyography (EMG) of the right FDI muscle were recorded with 9–mm–diameter Ag-AgCl surface electrodes. The active and reference electrodes were placed over the FDI muscle belly and metacarpophalangeal joint of the index finger, respectively. Ground electrode was placed on the dorsum of the hand. The signal was amplified (1000×), bandpass filtered (20 Hz −2.5 kHz; Intronix Technologies Model 2024F), digitized at 5 kHz by an analogue-to-digital interface (Micro1401; Cambridge Electronics Design, Cambridge, UK), and stored in a computer for off-line analysis using Signal 6.0 software.

### 2.4 Experimental setup

**Figure 1:**
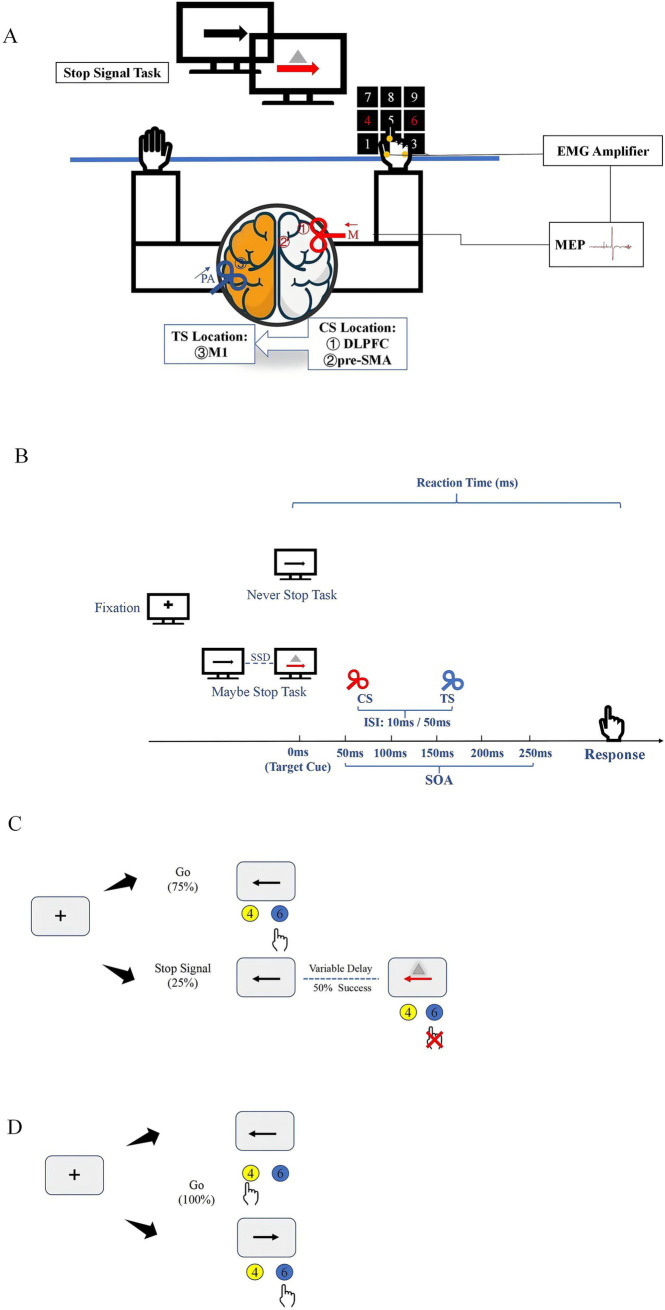
Experimental setup and tasks **(A)**. TMS coil sites and experimental setup. Participants sat in front of a computer in a relaxed position while different movement inhibition tasks were displayed on the screen. The CS coil (a small 50 mm figure-of-8 coil) was placed over the right hemisphere to induce medially (M) directed current in the brain (red arrow). The regions stimulated by CS coils were ① DLPFC and ② pre-SMA. The TS coil (a small 50 mm figure-of-8 coil) was applied over the hand representation of the left hemisphere to induce current in PA (posterior–anterior) direction in the brain (blue arrow). **(B)** Stimulus configurations used in study. **(C)** Outline of maybe stop task. The red arrow and gray triangle served as the visual stop signal. **(D)** Outline of never stop task. CS, conditioning stimulus; ISI, interstimulus intervals; SOA, stimulus-onset asynchrony; TS, test stimulus.

The TMS coils and stimulus configurations are shown in Figures 1A,B. Participants sat in a relaxed position, with their elbows, hips, and knees flexed at 90–100°, in front of a computer screen placed 75–85 cm away. TMS coils were applied to various locations over the left and right hemispheres while participants completed the SST, presented in random order through MATLAB software. A blank screen was presented for 3–4 s after each response and the inter-pulse intervals were > 5 s to avoid changes in motor excitability due to TMS. Although a variety of intensities have been used in previous studies of cortical interactions, the suprathreshold conditioning pulses can elicit functional interactions between the frontal cortex and M1 (Hasan et al., 2013). The interaction between the cortex is related to the ISI, and the different cortico-cortical interaction also shows different changes under the same ISI (Picazio et al., 2014). The more significant interactions at short- and long-latency between right motor-related regions and left M1 were observed at 10 ms and 50 ms (Ni et al., 2009; Cao et al., 2022). Therefore, we refer to our pervious study and set the CS intensity at 110% RMT, while ISI selected 10 ms and 50 ms to represent the short- and long-latency, respectively (Wei et al., 2024).

All participants underwent high-resolution T1 structural magnetic resonance imaging (MRI) using a three-dimensional fast spoiled gradient echo sequence (slice thickness: 1 mm; repetition time: 3130 ms; echo time: 2.98 ms; matrix: 256 × 256; field of view: 256; 176 sagittal slices) to localize individual right DLPFC and pre-SMA targets using the Visor 2 TMS Neuro-navigation system (eemagine, Berlin, Germany). Based on previous studies (Aron et al., 2007; Cao et al., 2022), the right DLPFC target was in Brodmann area (BA) 46 (Talairach coordinates: x = 40, y = 28, z = 30), and the pre-SMA target was in BA6 (Talairach coordinates: x = 6, y = 20, z = 44). The right DLPFC and pre-SMA stimulus locations were determined for each participant with the guidance of a coil tracker, and the CS coil was adjusted for each participant to ensure precise placement on the target gyrus.

### 2.5 Stop-signal task

In the SST, each trial began with the display of a black fixation cross on a white background for 500 ms, followed by a leftward or rightward arrow for 1,000 ms. Participants were instructed to respond as quickly and accurately as possible by pressing the “4” (leftward arrow) or “6” (rightward arrow) button on the keyboard using their right index finger.

The MST consisted of 75% go trials and 25% stop trials, with a total of 10 blocks, 600 trials (450 go trials and 150 stop trials). In the stop trials, a stop signal was presented by changing the arrow from black to red and displaying a gray triangle, after a variable stop-signal delay (SSD). To maintain a 50% stop success rate, a stepwise algorithm adjusted the SSD: it increased by 50 ms following a successful stop response and decreased by 50 ms after a failed stop response. The initial SSD was set to 250 ms. The TMS stimulus was applied at various stimulus-onset asynchronies (SOA) after the stop signal appeared (50 ms, 100 ms, 150 ms, 200 ms, 250 ms). For each block, three types of TMS stimulation (TS alone, 10 ms, and 50 ms) under different SOA were tested, with one trial of each stimulation collected. In the go trials, to prevent participants from guessing the trial type based on TMS, TMS was applied to all go trials. The TMS stimulus was applied at 50 ms, 100 ms, 150 ms, 200 ms, and 250 ms after the appearance of the go trials. Each block included three types of TMS stimulation (TS alone, 10 ms, and 50 ms) under different SOA, with three trials of each stimulation collected (Figure 1C).

The NST consisted of 180 go trials across three blocks, using the same stimuli as the MST go trials (Figure 1D). To avoid practice effect and reduce learning effect, the MST and NST were counterbalanced across participants.

### 2.6 Statistical analysis

The participants’ years of education and MoCA scores were not normally distributed, so the Mann–Whitney U test was used for analysis. Age was compared using a *t*-test, and sex differences were examined using a Chi-square test. Reaction times (RT) from go trials were screened for outliers, defined as responses that were incorrect or deviated from the mean by shorter or longer than the mean ± 3 standard deviations (SD), and these trials were excluded from further analysis. Proactive inhibition was assessed by calculating the RDE, defined as the difference between the go RT in the MST and NST (go RT__MST_ – go RT__NST_). Both the SSRT and RDE were analyzed using a two-way repeated-measures analysis of variance (RM ANOVA), with the group (PD, HC) as the between-subjects factor and the brain region (R DLPFC–L M1, pre-SMA–L M1) as the within-subject factor.

Peak-to-peak MEP amplitudes were extracted using a custom script in SIGNAL 6.0 (Cambridge Electronics Design, Cambridge, UK). Resting-state interhemispheric interactions were analyzed using a two-way RM ANOVA [group (PD, HC) × ISI (10 ms, 50 ms)] for the R DLPFC–L M1 and pre-SMA–L M1 MEP ratios. The effect of the R DLPFC–L M1 and pre-SMA–L M1 interaction for each participant was quantified as the ratio of the mean MEP amplitude in the paired-pulse conditions relative to that in the TS alone.

To measure R DLPFC–L M1 and pre-SMA–L M1 interactions during stopping, two separate three-way RM ANOVA were conducted, with group (PD, HC) as the between-subjects factor, and ISI (10 ms, 50 ms) and SOA (50, 100, 150, 200, 250 ms) as within-subject factors. TMS trials with background root mean square EMG within 2SD of the mean for 100 ms before the TMS pulse were included in the analysis. A similar three-way RM ANOVA (group: PD, HC; ISI: 10 ms, 50 ms; SOA: 50, 100, 150, 200, 250 ms) was applied to analyze the R DLPFC–L M1 and pre-SMA–L M1 interactions during go trials in the MST and NST conditions separately. Normality of the data distribution was assessed and confirmed using the Shapiro–Wilk test.

Planned polynomial contrasts were performed to identify the trend models that best explained the reactive inhibition performance of the two groups at different SOA. The linear, quadratic, and cubic models were tested for significance. The Greenhouse–Geisser method was used to correct for violations of sphericity. Post-hoc comparisons were conducted using paired *t*-tests. Statistical significance was set at *p* < 0.05, with Bonferroni correction for multiple comparisons. Additionally, Pearson correlation analysis was used to examine the relationship between the slope of change in MEP ratio and the SSRT and UPDRS–III scores during 150-200 ms after the appearance of the stop signal at 10 ms.

## 3 Results

The demographic and clinical data of the patients are summarized in Supplementary Table 1. As expected, no significant differences were found between patients with PD and HC in terms of age, sex, MoCA scores and educational level (Table 1).

**Table 1:** Comparison of demographic data in PD and HC.

	PD (*n* = 30)	HC (*n* = 30)	X^2^/t/z	p
Age	67 ± 4.51	66.6 ± 3.97	0.365	0.717
Sex (Male: Female)	17: 13	11: 19	2.411	0.121
Education (Years)	12.1 ± 2.63	12.1 ± 2.05	−0.016	0.987
MoCA	26.90 ± 0.92	27.4 ± 1.10	−1.832	0.062

Data is shown as mean ± standard deviation. HC, healthy control; MoCA, Montreal Cognitive Assessment; PD, Parkinson’s disease. The *t*-test (age), Chi-square test (sex) and Mann–Whitney U-tests (education, MoCA) were used to compare the differences between PD and HC.

### 3.1 Behavioral performance

**Figure 2:**
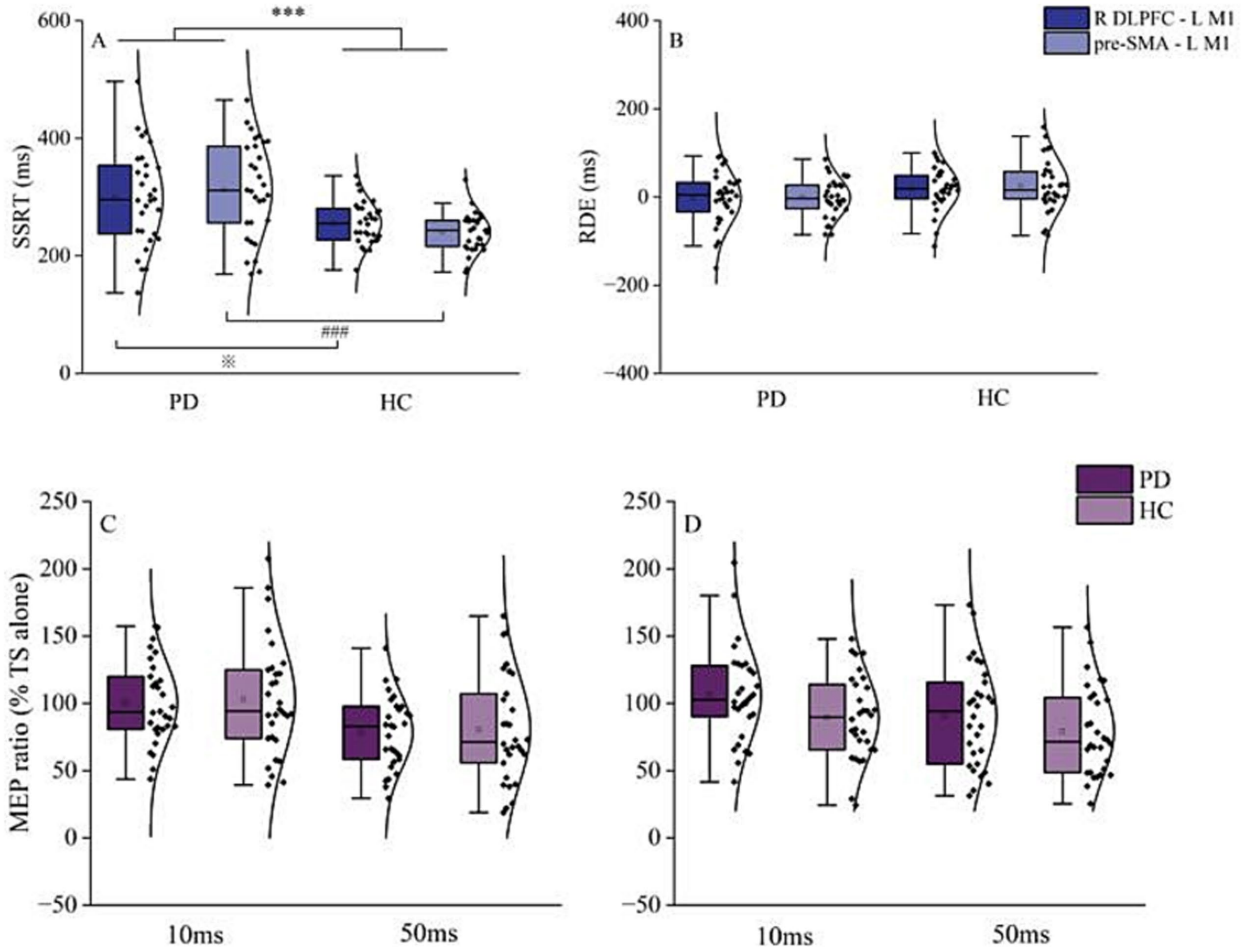
Behavioral and resting-state interhemispheric interaction results. **(A)** Results for reactive inhibition. SSRT in PD patients was significantly longer than that of HC, in both R DLPFC–L M1 and pre-SMA–L M1 conditions. **(B)** Results for proactive inhibition. There was no significant difference in RDE between PD patients and HC in the R DLPFC–L M1 and pre-SMA–L M1 conditions. **(C)** Interhemispheric interaction of R DLPFC–L M1 at ISI of 10 and 50 ms. **(D)** Interhemispheric interaction of pre-SMA–L M1 at ISI of 10 and 50 ms. Top and bottom lines of the box plot represent 1.5 times the interquartile range (IQR); the black horizontal line in the box plot indicates the median for each group; the black curve on the right shows the data distribution for each group. Each dot represents one subject. HC, healthy controls; ISI, interstimulus intervals; L M1, left primary motor cortex; PD, Parkinson’s disease; RDE, response delay effect; R DLPFC, right dorsal lateral prefrontal cortex; SSRT, stop-signal reaction time. ^***^p < 0.001, overall comparison of SSRT of PD and HC in R DLPFC–L M1 and pre-SMA–L M1; ^※^*p* < 0.05, R DLPFC–L M1 SSRT for PD vs. HC; ^###^*p* < 0.001, pre-SMA–L M1 SSRT for PD vs. HC.

Two-way RM ANOVA revealed a significant difference in SSRT between HC and patients with PD [*F* (1, 58) = 14.8, *p* < 0.001, η^2^_p_ = 0.204]. Although no main effect of region was observed, the interaction between region and group was significant [*F* (1, 58) = 4.9, *p* = 0.031, η^2^_p_ = 0.077]. Post-hoc paired *t*-tests showed that PD patients had significantly longer SSRT than HC for both R DLPFC–L M1 (*p* = 0.011) and pre-SMA–L M1 (*p* < 0.001) interactions (Figure 2A). Meanwhile, a two-way RM ANOVA on RDE revealed no significant effects for group, region, or group × region interactions (Figure 2B).

### 3.2 Resting state interhemispheric interactions

Paired-samples *t*-tests revealed no significant difference between PD patients and HC in R DLPFC–M1 or pre-SMA–L M1 interactions for MEP amplitudes from TS alone (Table 2). Two-way RM ANOVA showed that the main effect of ISI was significant for both R DLPFC–L M1 [*F* (1, 58) = 23.1, *p* < 0.001, η^2^_p_ = 0.284] and pre-SMA–L M1 interactions [*F* (1, 58) = 5.9, *p* = 0.018, η^2^_p_ = 0.093]. However, there was no significant effect of group and no significant group × ISI interaction (Figures 2C,D).

**Table 2:** MEP amplitudes from test stimulus alone in resting state in PD and HC groups.

	PD (*n* = 30)	HC (*n* = 30)
R DLPFC - L M1	0.76 ± 0.33 mV	0.88 ± 0.41 mV
pre-SMA - L M1	0.85 ± 0.46 mV	0.91 ± 0.47 mV

L, left; R, right. Values are shown as mean ± standard deviation.

### 3.3 R DLPFC–L M1 interhemispheric interaction during motor performance

In the go/stop trials of both MST and NST, MEP amplitudes for TS alone at each SOA did not differ between groups (Supplementary Table 2). For the R DLPFC–L M1 interaction, three-way RM ANOVA revealed significant main effects of ISI [*F* (1, 58) = 688.2, *p* < 0.001, η^2^_p_ = 0.922], SOA [*F* (4, 58) = 20.6, *p* < 0.001, η^2^_p_ = 0.262], and group [*F* (1, 58) = 7.8, *p* = 0.007, η^2^_p_ = 0.119] during stopping. A significant group × ISI × SOA interaction was detected [*F* (4, 58) = 2.7, *p* = 0.033, η^2^_p_ = 0.044].

**Figure 3:**
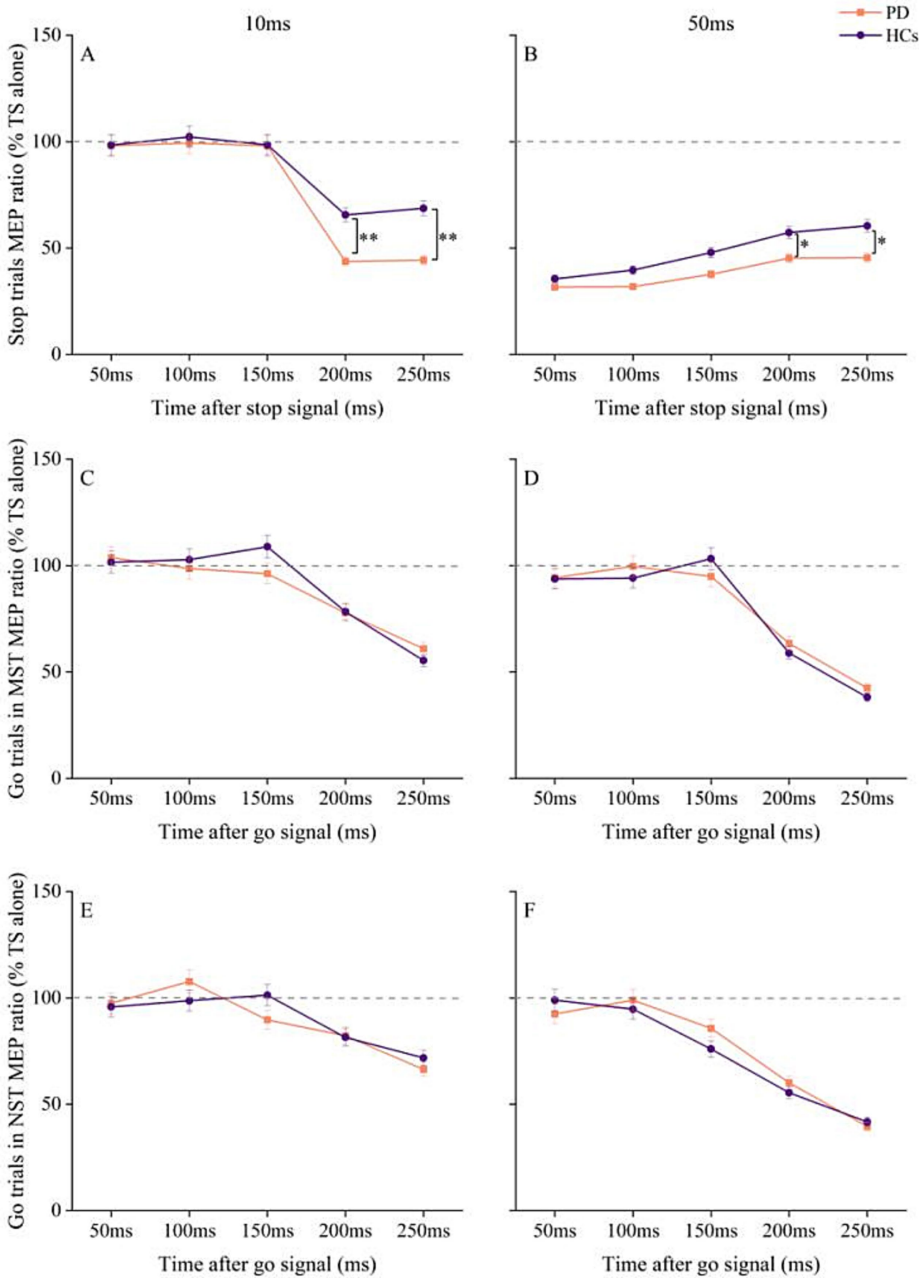
Changes in R DLPFC–L M1 interactions with time after stop and go signals under different tasks conditions in PD and HC. **(A)** At ISI of 10 ms, changes in R DLPFC–LM1 interaction at different times after the appearance of stop signal. **(B)** At ISI of 50 ms, changes in R DLPFC–L M1 interaction at different times after the appearance of stop signal. **(C)** R DLPFC - L M1 interaction at ISI of 10 ms for go trials at different times after the go signal in the maybe stop condition. **(D)** R DLPFC–L M1 interaction at ISI of 50 ms for go trials at different times after the go signal in the maybe stop condition. **(E)** R DLPFC–L M1 interaction at ISI of 10 ms for go trials at different times after the go signal in the never stop condition. **(F)** R DLPFC–L M1 interaction at ISI of 50 ms for go trials at different times after the go signal in the never stop condition. The gray dashed lines indicate the MEP amplitude generated by TS alone (100%). Values below 100% represent inhibition and values above 100% represent facilitation. Values are shown as mean with standard deviation. HC, healthy controls; ISI, interstimulus intervals; MST, maybe stop task; NST, never stop task; PD, Parkinson’s disease; *p < 0.05, **p < 0.01, patients with PD vs. HC.

At an ISI of 10 ms, the R DLPFC–L M1 interaction showed more inhibition in PD patients at SOA of 200 ms and 250 ms relative to 50 ms, 100 ms, and 150 ms (*p* < 0.001). HC also showed greater inhibition at 200 ms and 250 ms compared to 50 ms, 100 ms, and 150 ms (*p* < 0.001). Post-hoc paired *t*-tests revealed greater inhibition in PD patients compared to HC at SOA of 200 ms (*p* = 0.001) and 250 ms (*p* = 0.002) (Figure 3A).

In contrast, at ISI of 50 ms, after Bonferroni correction for multiple SOA, the MEP ratios from stop trials in HC were significantly higher at SOA of 200 ms and 250 ms compared to 50 ms and 100 ms (*p* < 0.001). In PD patients, the MEP ratios during stopping were also higher at 200 ms and 250 ms compared to 50 ms and 100 ms (*p* < 0.05). Post-hoc analysis using paired *t*-test showed that interhemispheric disinhibition was more pronounced in HC than in PD patients at SOA of 200 ms (*p* = 0.038) and 250 ms (*p* = 0.045) (Figure 3B).

RM ANOVA for MEP ratios in go trials of MST showed significant main effects of ISI [*F* (1, 58) = 37, *p* < 0.001, η^2^_p_ = 0.389] and SOA [*F* (4, 58) = 85.3, *p* < 0.001, η^2^_p_ = 0.595], as well as an ISI × SOA interaction [*F* (4, 58) = 7.0, *p* < 0.001, η^2^_p_ = 0.108], but no significant effect of group or group × ISI × SOA interaction (Figures 3C,D). Similar results were observed for go trials in NST, with significant main effects of ISI [*F* (1, 58) = 49.1, *p* < 0.001, η^2^_p_ = 0.459] and SOA [*F* (4, 58) = 40.7, *p* < 0.001, η^2^_p_ = 0.412], as well as an ISI × SOA interaction [*F* (4, 58) = 9.9, *p* < 0.001, η^2^_p_ = 0.146], but no group differences or group × ISI × SOA interaction (Figures 2E,F).

Trend model analysis showed that the relationship between interhemispheric interaction at 10 ms ISI and SOA was modeled by a linear trend during reactive inhibition in both PD patients [*F* (1, 29) = 260.7, *p* < 0.001] and HC [*F* (1, 29) = 30.8, *p* < 0.001]. The MEP ratio of R DLPFC–L M1 interaction declined with increasing SOA in the PD group at both quadratic [*F* (1, 29) = 26.7, *p* < 0.001] and tertiary [*F* (1, 29) = 28.3, *p* < 0.001] models. At ISI of 50 ms, the relationship between R DLPFC–L M1 interaction and SOA showed a significant linear trend for both HC [*F* (1, 29) = 36.5, *p* < 0.001] and PD [*F* (1, 29) = 24, *p* < 0.001], suggesting that the MEP ratio of R DLPFC–L M1 interaction increased linearly with longer SOA in both groups.

### 3.4 Pre-SMA–L M1 interhemispheric interaction during motor performance

**Figure 4:**
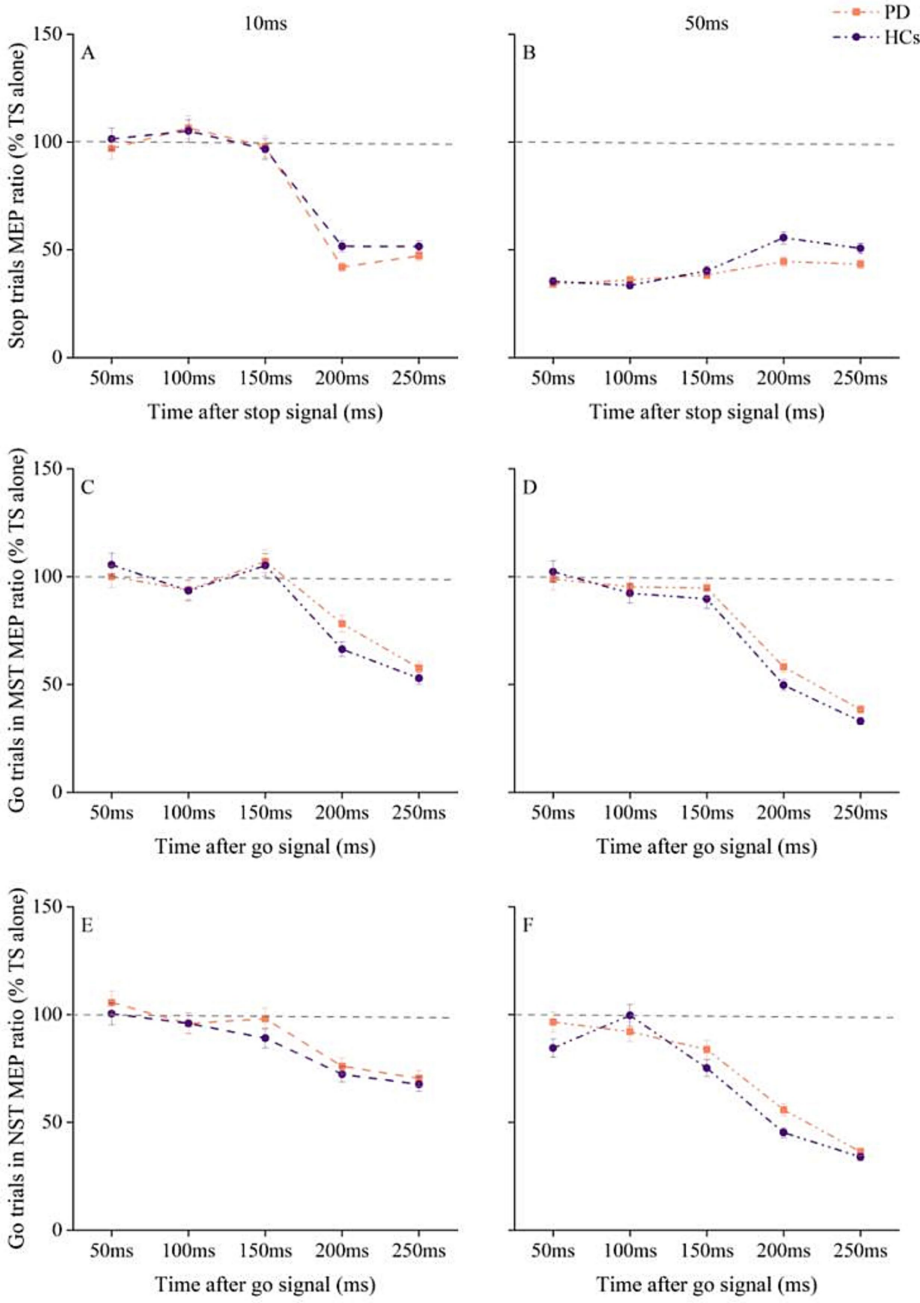
Changes in pre-SMA - L M1 interaction with time after stop and go signals under different tasks conditions in PD patients and HC. **(A)** Pre-SMA–L M1 interaction at ISI of 10 ms at different times after stop cue onset. **(B)** Pre-SMA–L M1 interaction at ISI of 50 ms at different times after stop cue onset. **(C)** pre-SMA–L M1 interaction at ISI of 10 ms for go trials in the maybe stop condition. **(D)** pre-SMA–L M1 interaction at ISI of 50 ms for go trials in the maybe stop condition. **(E)** pre-SMA–L M1 interaction at ISI of 10 ms for go trials in the never stop condition. **(F)** pre-SMA–L M1 interaction at ISI of 50 ms for go trials in the never stop condition. The gray dashed lines indicate the MEP amplitude generated by TS alone (100%). Values below 100% represent inhibition and values above 100% represent facilitation. Values are shown as mean with standard error. HC, healthy controls; ISI, interstimulus intervals; MST, maybe stop task; NST, never stop task; PD, Parkinson’s disease.

MEP amplitudes for TS alone of each SOA did not differ between groups for pre-SMA–L M1 interaction (Supplementary Table 2). Three-way RM ANOVA for pre-SMA–L M1 interaction showed a significant main effect for ISI [*F* (1, 58) = 729.7, *p* < 0.001, η^2^p = 0.926] and SOA [*F* (4, 58) = 41.2, *p* < 0.001, η^2^_p_ = 0.415], as well as a significant ISI × SOA interaction [*F* (4, 58) = 148.9, *p* < 0.001, η^2^_p_ = 0.720]. However, there was no significant effect of group and no significant group × ISI × SOA interaction (Figures 4A,B).

RM ANOVA for MEP ratios from MST go trials showed significant main effects of ISI [*F* (1, 58) = 61, *p* < 0.001, η^2^_p_ = 0.513]and SOA [*F* (4, 58) = 93.1, *p* < 0.001, η^2^_p_ = 0.616], and a significant ISI × SOA interaction [*F* (4, 58) = 9.9, *p* < 0.001, η^2^_p_ = 0.146], with no significant effect of group and no significant group × ISI × SOA interaction (Figures 4C,D).

We also observed significant main effects of ISI [*F* (1, 58) = 130.2, *p* < 0.001, η^2^_p_ = 0.692] and SOA [*F* (4, 58) = 57, *p* < 0.001, η^2^_p_ = 0.496], and a significant ISI × SOA interaction [*F* (4, 58) = 11.4, *p* < 0.001, η^2^_p_ = 0.164] for pre-SMA–L M1 interaction during the NST. However, there were no significant effects of group and no significant group × ISI × SOA interaction (Figures 4E,F).

### 3.5 Interhemispheric interaction correlates with SSRT and UPDRS–III in PD patients

**Figure 5:**
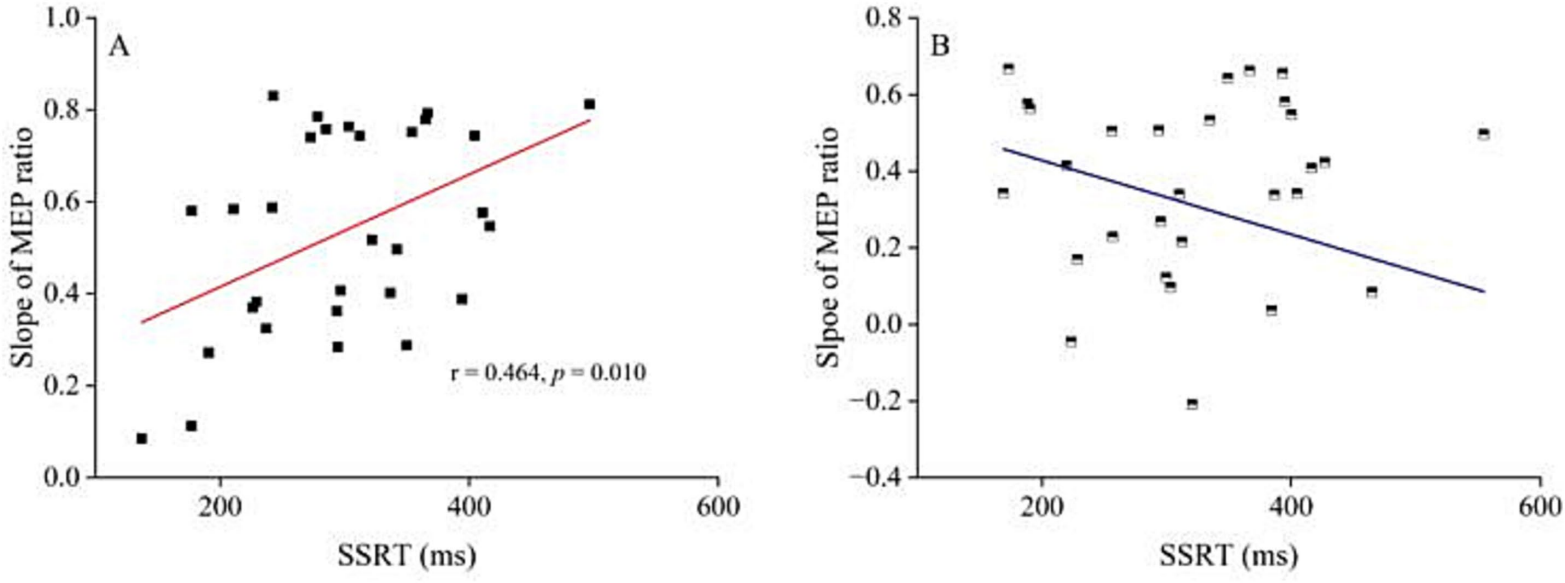
The relationship between R DLPFC–L M1 interaction at interstimulus interval of 10 ms and stop-signal reaction time. **(A)** The relationship between the decline slope of MEP ratio _R DLPFC - L M1_ after stop signal at 150–200 ms and SSRT for ISI of 10 ms in PD patients. Pearson correlation analysis showed significant correlation between the slope of R DLPFC–L M1 interaction decline and SSRT (*r* = 0.464, *p* = 0.010). **(B)** The relationship between the decline slope of MEP ratio _R DLPFC - L M1_ at 150–200 ms after stop signal at ISI of 10 ms and SSRT in HC. Pearson correlation analysis showed no correlation between the slope of R DLPFC–L M1 interaction decline and SSRT. HC, healthy controls; ISI, interstimulus intervals; PD, Parkinson’s disease.

Since the trend analysis indicated that the inhibitory interaction of R DLPFC–L M1 in PD patients at the ISI of 10 ms began to decline sharply at 150 ms, with significant differences from HC at 200 ms. To investigate whether this sharp decline is associated with behavioral performance, we first calculated the slope change in the difference from 150 ms to 200 ms relative to the 150 ms ratio. The slope was calculated using the formula: (MEP ratio150ms − MEP ratio200ms) / MEP ratio150ms, which was updated after outlined methods by Wei et al. (2024). We then analyzed the relationship between the slope changes and SSRT and UPDRS–III. Pearson correlation analysis revealed that SSRT was positively correlated with the slope of the MEP ratio for PD patients (*r* = 0.464, *p* = 0.010), but not for HC (Figure 5). Furthermore, there was no correlation between the slope changes in MEP ratio for R DLPFC–L M1 interaction and UPDRS–III at 150–200 ms.

## 4 Discussion

We investigated the cortical interactions between the right DLPFC and pre-SMA to left M1 in PD patients during stop task performance. The results showed that SSRT was significantly slower in PD patients compared to HC. Additionally, the inhibition interaction of R DLPFC–L M1 was greater at both 10 ms and 50 ms during the stop task. Specifically, when the ISI was 10 ms, the MEP ratio for the R DLPFC–L M1 interaction between 150–200 ms after the stop cue onset was positively correlated with SSRT in PD patients. No significant differences in pre-SMA–L M1 interaction were found between PD patients and HC.

### 4.1 PD affected mainly reactive inhibition

The significantly prolonged SSRT observed in PD patients in this study reflects impaired reactive inhibition, consistent with our previous findings (Wang et al., 2022a). This deficit may be attributed to the degeneration of dopaminergic neurons, which leads to alterations in the frontal–basal ganglia circuit (MacDonald et al., 2000; Miller and Cohen, 2001). In PD patients, motor inhibition signals from the BG are diminished, impairing the ability to reduce the competition between movement and desired inhibition (Mink, 1996). However, no significant differences were observed between the groups in RDE, which may be related to the stage of the disease. A study showed that individuals with early PD have difficulty completely stopping an action compared to controls, but they can still adjust their behavioral strategies based on contextual cues. Additionally, the findings may be influenced by the cognitive demands of the task. Unlike NST, the MST requires participants to monitor and respond to both go and stop signals, increasing cognitive load. Proactive inhibition is influenced by a combination of cognitive processing and motor ability (Wang et al., 2022b). As the patients in our study had normal cognitive function, this may explain the normal levels of proactive inhibition observed in our PD participants.

### 4.2 Interhemispheric interaction in resting state did not differ between PD patients and HC

We found no differences in interhemispheric interaction at rest between PD patients and HC at both short and long ISI (10 ms and 50 ms). These circuits may not be active during the resting state, and interhemispheric interactions could be driven by dynamic movements, as neural representations are influenced by a combination of external stimuli and internal biases (Nachev et al., 2007). In a study of subcortical stroke, connectivity between motor areas at rest was found to be normal, as assessed by functional magnetic resonance imaging (fMRI) (Grefkes et al., 2008). However, during voluntary movements with the paretic limbs, significant differences in interhemispheric connectivity were observed compared to HC (Rehme et al., 2011; Volz et al., 2015).

### 4.3 R DLPFC–L M1 interaction in PD patients showed excessive inhibition at ISI of 10 ms

At 10 ms ISI, MEP ratios for the R DLPFC–L M1 interaction were lower at 200 and 250 ms after the stop signal in both PD and HC compared to earlier time points, with greater inhibition observed in PD patients. Motor inhibition is associated with increased cortical inhibition (Julie Duque et al., 2017). Approximately 200 ms is needed from the onset of the stop signal to movement cancellation (Derosiere and Duque, 2020). The DLPFC is linked to the successful inhibition of incompatible actions, resolving competition and establishing the mappings necessary to perform the task, converting conceptual information into action (Miller and Cohen, 2001; Duque et al., 2012; Lucci et al., 2014). Specifically, the right DLPFC plays a crucial role in the process of reactive inhibition (Chen et al., 2021). The BG-mediated inhibition network integrates multiple sources of information from frontal regions, including the DLPFC, and passes this integrated information to M1 to determine the most appropriate decision and execute it (Miyachi et al., 2005; Trujillo et al., 2019). However, functional impairment in the BG leads to a loss of motor cortex selectivity, affecting the activity of the cortical inhibitory system (Bares et al., 2003). Additionally, PD patients exhibit decreased regional cerebral blood flow in the right DLPFC due to pathological factors (Kikuchi et al., 2001). Dopamine deficits reduce cortical activity (Fecteau et al., 2007; Kehagia et al., 2012), which disrupts the activation of the right DLPFC during task performance in PD patients (Disbrow et al., 2013; Loayza et al., 2022). Impaired neurotransmitter system activation or disrupted connectivity between regions can disrupt the balance between excitatory and inhibitory processes in the brain, further affecting the integration of sensory information necessary for inhibitory actions (Langan et al., 2010; Passamonti et al., 2012; Kapogiannis et al., 2013). Consequently, the regulation of the right DLPFC over M1 during automatic movement is compromised in PD, potentially leading to deficits in reactive inhibition in PD patients. Suppression of activities in the R DLPFC using cathodal transcranial direct current stimulation (tDCS) improved response inhibition capacity (Weidacker et al., 2016).

### 4.4 R DLPFC–L M1 interaction in PD patients showed insufficient disinhibition at ISI of 50 ms

We observed distinct changes in interhemispheric interaction at 50 ms ISI. The MEP ratio in HC showed an increasing trend of disinhibition during stopping, whereas a weaker trend was observed in PD patients, particularly at 200 and 250 ms after the stop cue onset. This finding may be attributed to abnormal mediating activity in interhemispheric transmission in PD patients. Although the right DLPFC and left M1 lack a direct anatomical connection due to the lack of white matter fiber connections (Guye et al., 2003), evidence suggests conduction via relays in homologous M1 at long latencies (Ni et al., 2009). Abnormal interhemispheric M1–M1 interactions in PD may impede the right DLPFC-left M1 interhemispheric nerve conduction, affecting the regulation of the DLPFC on the motor system. This is reflected as sustained inhibition of cortico-cortical interaction. Furthermore, interhemispheric interactions transmitted through the corpus callosum (CC) are glutamate-dependent and typically excitatory (disinhibitory) (van der Knaap and van der Ham, 2011). Previous studies have shown that interhemispheric disinhibition through the DLPFC projection via the CC to the contralateral M1 enhances bimanual performance in the elderly (Fujiyama et al., 2016). However, in PD patients, pathological damage to CC fibers occurs in the early stages of the disease (Wittstock, 2009), potentially leading to insufficient disinhibition and poor motor performance during the task.

### 4.5 Pre-SMA–L M1 interaction did not affect reactive inhibition in PD patients

Although the MEP ratios for pre-SMA–L M1 interaction showed inhibition during stopping, we found no significant group differences. The pre-SMA interacts with M1 to regulate planned motor adaptation in response to environmental stimuli (Nachev et al., 2008; Rossi et al., 2009; Neubert et al., 2010). This behavioral modulation, occurring when the participant anticipates a stop signal, is a manifestation of proactive inhibition (Obeso et al., 2013). The SST findings suggest that pre-SMA activity reflects the motivation to regulate actions (Scangos and Stuphorn, 2010) and is associated with preparation-related activity (Chikazoe et al., 2009; Jahfari et al., 2010).

### 4.6 Excessive R DLPFC–L M1 interhemispheric interaction predicts deficits in reactive inhibition in PD patients

Our results show that a greater decline in the slope of the MEP ratio for _R DLPFC–L M1_ correlated with slower SSRT at 150–200 ms after the stop signal onset at an ISI of 10 ms. Interhemispheric interaction is associated with behavioral performance (van Ruitenbeek et al., 2017). Decreased DLPFC–M1 interaction not only affects the execution of self-initiated movements (Wu et al., 2011b), but is also associated with decreased bimanual coordination in older adults (Fujiyama et al., 2016). The DLPFC is anatomically connected to the pre-SMA, subthalamic nucleus, and other regions, with these polysynaptic connections providing multiple pathways for the DLPFC to modulate M1 excitability (Brown et al., 2019). However, the regulatory effect of the right DLPFC on the M1 during automatic movement is affected in PD (François-Brosseau et al., 2009). Therefore, we suggest that excessive inhibition at 150–200 ms after stop-signal onset may be a physiological manifestation of reduced movement inhibition deficits in PD patients, and changes in brain interaction regulation during the task can be regarded as a signal of behavioral performance (Hinder et al., 2012).

Our study has limitations. Although we used coordinates to identify the target stimulating brain region for each participant, individual brain differences may introduce some biases. In future studies, we will use effective field modeling to improve accuracy. Additionally, due to the limited number of participants in the early stage of recruitment, this study has not thoroughly analyzed the impact of clinical peculiarities on electrophysiology and behavior. Future studies will explore clinical symptoms such as disease duration, gender, age, cognitive level, and other factors, recruit more PD patients at different stages, and further explore the relationship between clinical variability and neurophysiology and its impact on behavioral performance. Moreover, combined with fMRI and TMS-EEG, we will explore the impact of interactions between SMC and other regions on motor preparation, initiation and execution in PD patients.

## 5 Conclusion

To sum up, this study used dual-site ppTMS for the first time to examine PD patients during SST performance. Impaired reactive inhibition in PD patients may be associated with abnormal regulation of R DLPFC–L M1 inhibition, which exhibited excessive inhibition at both short (10 ms) and long (50 ms) ISIs during stopping. The degree of inhibition interaction at short ISI was found to correlate with stopping efficiency, as measured by SSRT. This abnormal interhemispheric interaction may provide a physiological correlation for some of the behavioral deficits observed in PD patients.

## Data Availability

The raw data supporting the conclusions of this article will be made available by the authors, without undue reservation.

## Glossary

CS: Conditioning stimulus
DLPFC: Dorsolateral prefrontal cortex
FDI: First dorsal interosseous
fMRI: Functional magnetic resonance imaging
HC: Healthy controls
ISI: Interstimulus intervals
L: Left
M1: Primary motor cortex
MEPs: Motor evoked potentials
MST: Maybe stop task
MoCA: Montreal Cognitive Assessment
NST: Never stop task
PD: Parkinson’s disease
pre-SMA: Pre-supplementary motor area
ppTMS: Paired-pulse transcranial magnetic stimulation
RT: Reaction time
R: Right
RDE: Response delay effect
RMT: Resting motor threshold
SSRT: Stop-signal reaction time
SST: Stop-signal task
SOA: Stimulus–onset asynchrony
SSD: Stop signal delay
SMC: Supplementary motor complex
EMG: Surface electromyography
TMS: Transcranial magnetic stimulation
tDCS: Transcranial direct current stimulation
TS: Test stimulus
MDS–UPDRS–III: Movement Disorder Society-Unified Parkinson’s Disease Rating Scale-Part III
