# Supplementary material for: Role of right dorsolateral prefrontal cortex–left primary motor cortex interaction in motor inhibition in Parkinson’s disease
Source: Front Aging Neurosci. 2025 Mar 5;17:1524755. doi: 10.3389/fnagi.2025.1524755 (PMC11919838; doi:10.3389/fnagi.2025.1524755)
Supplement: Supplementary file 1 [file Table_1.docx]

**Supplement Table 1.** Demographic and clinical data in Parkinson’s disease group.

| **Patient** | **Sex** | **Age (years)** | **Disease duration (years)** | **Hoehn &Yahr stage** | **UPDRS-III** | **LEDD (mg)** | **MoCA** | **More affected side** |
| --- | --- | --- | --- | --- | --- | --- | --- | --- |
| 1 | F | 63 | 4 | 1 | 15 | 203 | 28 | R |
| 2 | M | 70 | 4 | 1 | 13 | 300 | 28 | R |
| 3 | M | 65 | 7 | 1 | 32 | 375 | 26 | L |
| 4 | F | 70 | 6 | 1 | 11 | 675 | 28 | R |
| 5 | M | 68 | 3 | 1 | 12 | 225 | 26 | R |
| 6 | F | 67 | 5 | 1 | 14 | 278 | 27 | L |
| 7 | M | 75 | 6 | 2 | 35 | 650 | 27 | R |
| 8 | M | 75 | 7 | 2 | 28 | 300 | 27 | L |
| 9 | F | 61 | 3 | 1 | 29 | 175 | 26 | L |
| 10 | F | 68 | 3 | 1 | 26 | 338 | 26 | L |
| 11 | M | 69 | 7.5 | 2 | 32 | 475 | 27 | L |
| 12 | M | 72 | 7 | 1 | 24 | 450 | 26 | R |
| 13 | F | 64 | 2 | 1 | 6 | 699 | 27 | L |
| 14 | M | 69 | 7 | 1 | 11 | 308 | 27 | R |
| 15 | F | 68 | 3 | 1 | 23 | 150 | 26 | L |
| 16 | M | 67 | 13 | 2 | 29 | 450 | 28 | R |
| 17 | M | 63 | 4 | 1 | 8 | 450 | 26 | L |
| 18 | F | 65 | 8 | 1 | 14 | 375 | 27 | L |
| 19 | F | 69 | 3 | 2 | 27 | 600 | 27 | L |
| 20 | M | 63 | 6 | 2 | 49 | 1207 | 27 | L |
| 21 | M | 64 | 4 | 1 | 16 | 675 | 26 | R |
| 22 | F | 64 | 8 | 1 | 20 | 475 | 26 | L |
| 23 | M | 72 | 5 | 2 | 20 | 400 | 29 | R |
| 24 | F | 75 | 5 | 1 | 5 | 426 | 28 | R |
| 25 | M | 62 | 3 | 1 | 12 | 288 | 27 | L |
| 26 | M | 70 | 3 | 1 | 20 | 188 | 29 | L |
| 27 | M | 65 | 3 | 1 | 10 | 475 | 26 | L |
| 28 | F | 60 | 3 | 1 | 14 | 425 | 27 | L |
| 29 | F | 57 | 2 | 1 | 9 | 188 | 26 | R |
| 30 | M | 70 | 6 | 1 | 21 | 238 | 26 | L |

F: female; L: left; LEDD, levodopa equivalent daily dose; M: male; MoCA, Montreal Cognitive Assessment; PD, R: right; UPDRS-III, Unified Parkinson’s Disease Rating Scale, Part III (on-medication).

**Supplement Table 2.** MEP amplitudes from test stimulus alone in MST and NST trials for PD and HC.

| **Trial type** | **SOA (ms)** | **R DLPFC - L M1 (mV)** | | | **pre-SMA - L M1 (mV)** | | |
| --- | --- | --- | --- | --- | --- | --- | --- |
|  |  | **PD(n = 30)** | **HC(n = 30)** | | **PD(n = 30)** | | **HC(n = 30)** |
| **Stop** | **50** | 0.93 ± 0.20 | | 1.00 ± 0.19 | 0.98 ± 0.15 | 0.98 ± 0.22 | |
|  | **100** | 1.08 ± 0.11 | | 1.01 ± 0.18 | 1.04 ± 0.15 | 1.07 ± 0.16 | |
|  | **150** | 1.13 ± 0.15 | | 1.13 ± 0.19 | 1.14 ± 0.15 | 1.11 ± 0.21 | |
|  | **200** | 1.00 ± 0.18 | | 1.01 ± 0.18 | 0.96 ± 0.16 | 0.94 ± 0.18 | |
|  | **250** | 0.86 ± 0.18 | | 0.84 ± 0.24 | 0.91 ± 0.16 | 0.89 ± 0.24 | |
| **MST Go** | **50** | 0.80 ± 0.20 | | 0.78 ± 0.25 | 0.78 ± 0.15 | 0.71 ± 0.24 | |
|  | **100** | 0.81 ± 0.20 | | 0.74 ± 0.21 | 0.84 ± 0.17 | 0.78 ± 0.23 | |
|  | **150** | 0.90 ± 0.12 | | 0.80 ± 0.18 | 0.79 ± 0.12 | 0.77 ± 0.19 | |
|  | **200** | 1.07 ± 0.15 | | 1.13 ± 0.21 | 1.11 ± 0.15 | 1.19 ± 0.19 | |
|  | **250** | 1.43 ± 0.36 | | 1.55 ± 0.38 | 1.48 ± 0.28 | 1.56 ± 0.39 | |
| **NST Go** | **50** | 0.80 ± 0.22 | | 0.78 ± 0.30 | 0.77 ± 0.26 | 0.76 ± 0.31 | |
|  | **100** | 0.80 ± 0.22 | | 0.76 ± 0.31 | 0.86 ± 0.27 | 0.66 ± 0.28 | |
|  | **150** | 0.87 ± 0.23 | | 0.85 ± 0.25 | 0.85 ± 0.20 | 0.78 ± 0.24 | |
|  | **200** | 1.05 ± 0.21 | | 1.08 ± 0.35 | 1.09 ± 0.28 | 1.20 ± 0.21 | |
|  | **250** | 1.47 ± 0.37 | | 1.54 ± 0.47 | 1.44 ± 0.38 | 1.60 ± 0.46 | |

MST, Maybe Stop Task; NST, Never Stop Task; SOA, Stimulus-Onset Asynchrony. Values are shown as mean ± standard deviation.
